# Epidemiology and analysis of SARS-CoV-2 Omicron subvariants BA.1 and 2 in Taiwan

**DOI:** 10.1038/s41598-023-43357-7

**Published:** 2023-10-03

**Authors:** Li-Teh Liu, Shyh-Shin Chiou, Po-Chih Chen, Chun-Hong Chen, Ping-Chang Lin, Ching-Yi Tsai, Wan-Long Chuang, Shang-Jyh Hwang, Inn-Wen Chong, Jih-Jin Tsai

**Affiliations:** 1 Department of Medical Laboratory Science and Biotechnology, College of Medical Technology, Chung Hwa University of Medical Technology, Tainan City, Taiwan; 2 Graduate Institute of Clinical Medicine, College of Medicine, Kaohsiung Medical University, Kaohsiung City, Taiwan; 3 Center of Applied Genomics, Kaohsiung Medical University, Kaohsiung City, Taiwan; 4 Division of Pediatric Hematology and Oncology, Department of Pediatrics, Kaohsiung Medical University Hospital, Kaohsiung City, Taiwan; 5 Department of Laboratory Medicine, Kaohsiung Medical University Hospital, Kaohsiung City, Taiwan; 6 Department of Medical Laboratory Science and Biotechnology, Kaohsiung Medical University, Kaohsiung City, Taiwan; 7 National Mosquito-Borne Diseases Control Research Center, National Health Research Institutes, Zhunan, Miaoli County Taiwan; 8 National Institute of Infectious Diseases and Vaccinology, National Health Research Institutes, Zhunan, Miaoli County Taiwan; 9 Tropical Medicine Center, Kaohsiung Medical University Hospital, Kaohsiung City, Taiwan; 10 School of Medicine, College of Medicine, Kaohsiung Medical University, Kaohsiung City, Taiwan; 11 Hepatobiliary Division, Department of Internal Medicine, Kaohsiung Medical University Hospital, Kaohsiung City, Taiwan; 12 Division of Nephrology, Department of Internal Medicine, Kaohsiung Medical University Hospital, Kaohsiung City, Taiwan; 13 Department of Internal Medicine and Graduate Institute of Medicine, Kaohsiung Medical University, Kaohsiung City, Taiwan; 14 Department of Pulmonary Medicine, Kaohsiung Medical University Hospital, Kaohsiung City, Taiwan; 15 Division of Infectious Diseases, Department of Internal Medicine, Kaohsiung Medical University Hospital, No. 100, Tzyou 1st Road, Kaohsiung City, 80756 Taiwan

**Keywords:** Microbiology, Diseases

## Abstract

The Omicron variant of severe acute respiratory syndrome coronavirus 2 (SARS-CoV-2), first detected in October 2021, possessed many mutations compared to previous variants. We aimed to identify and analyze SARS-CoV-2 Omicron subvariants among coronavirus disease 2019 (COVID-19) patients between January 2022 and September 2022 in Taiwan. The results revealed that BA.2.3.7, featuring K97E and G1251V in the spike protein compared with BA.2, emerged in March 2022 and persistently dominated between April 2022 and August 2022, resulting in the largest COVID-19 outbreak since 2020. The accumulation of amino acid (AA) variations, mainly AA substitution, in the spike protein was accompanied by increasing severity in Omicron-related COVID-19 between April 2022 and January 2023. Older patients were more likely to have severe COVID-19, and comorbidity was a risk factor for COVID-19-related mortality. The accumulated case fatality rate (CFR) dropped drastically after Omicron variants, mainly BA.2.3.7, entered Taiwan after April 2022, and the CFR was 0.16% in Taiwan, which was lower than that worldwide (0.31%) between April 2021 and January 2023. The relatively low CFR in Omicron-related COVID-19 patients can be attributed to adjustments to public health policies, promotion of vaccination programs, effective antiviral drugs, and the lower severity of the Omicron variant.

## Introduction

A lethal human-to-human transmitted severe respiratory disease, later known as coronavirus disease 2019 (COVID-19) was identified in December 2019^1^. Asymptomatic infection or different severities of COVID-19 develops after inhalation of droplets containing severe acute respiratory syndrome coronavirus 2 (SARS-CoV-2)^2,3^. SARS-CoV-2 has been detected in clinical samples such as nasopharyngeal swabs, sputum, blood, urine, and feces^4,5^. COVID-19 was declared a global pandemic disease by the World Health Organization (WHO) on March 11, 2020^6^. There have been 767 million confirmed cases of COVID-19 and 6.9 million COVID-19-related deaths globally as of June 2023 (https://covid19.who.int/) since then. Various variants of concern (VOCs) have been identified and are circulating worldwide and accumulating genetic mutations. A systematic review by Arabi et al.^7^ recently suggested that several studies have documented a higher transmissibility, milder symptoms, and a notable decrease in the likelihood of hospitalizations, intensive care unit (ICU) admissions, the need for oxygenation/ventilation, and mortality among individuals infected with the Omicron variant, in contrast to those infected with other variants such as Delta. Nevertheless, certain studies have indicated similar levels of severity in Omicron-infected patients when compared to other variants, underscoring a significant risk of severe illness^8–10^.

A series of public health measures by the Taiwan government (such as border control, real-time diagnosis, contact tracing, safe social distancing, mask-wearing, and timely and appropriate medical measures for severe COVID-19 patients) controlled the COVID-19 epidemic, resulting in 823 confirmed cases and 9 COVID-19-related deaths, with a case fatality rate (CFR) of 1.09% and without a complete lockdown in 2020^11^. However, a COVID-19 outbreak related to the Alpha variant featuring Spike D614G + M1237I occurred between May 2021 and June 2021, the time the COVID-19 vaccination program had just begun and the herd immunity was low in the community, hit Taiwan resulting in 13,795 confirmed COVID-19 cases and 820 COVID-19-related deaths, with a 5.95% CFR, which was higher than the global CFR (2.15%) in that period^12^. The variant that caused the COVID-19 outbreak in Taiwan was the same that had appeared worldwide, although slightly delayed. Taiwan survived the Delta period^13^ that came immediately after the previous outbreak related to the Alpha variant, but the emerging Omicron variant quietly entered Taiwan at the end of 2021.

In this study, we collected the clinical samples from the nasopharyngeal swabs and identified Omicron subvariants among patients suspected of COVID-19 between January 2022 and September 2022. The sequences of the genome or spike gene were analyzed. We performed phylogenetic and clade replacement analyses to determine the dominant Omicron subvariants in the outbreak in Taiwan. The severity and CFR were further analyzed.

## Results

### Prevalence of Omicron subvariants between December 2021 and January 2023 in Taiwan

**Figure 1:**
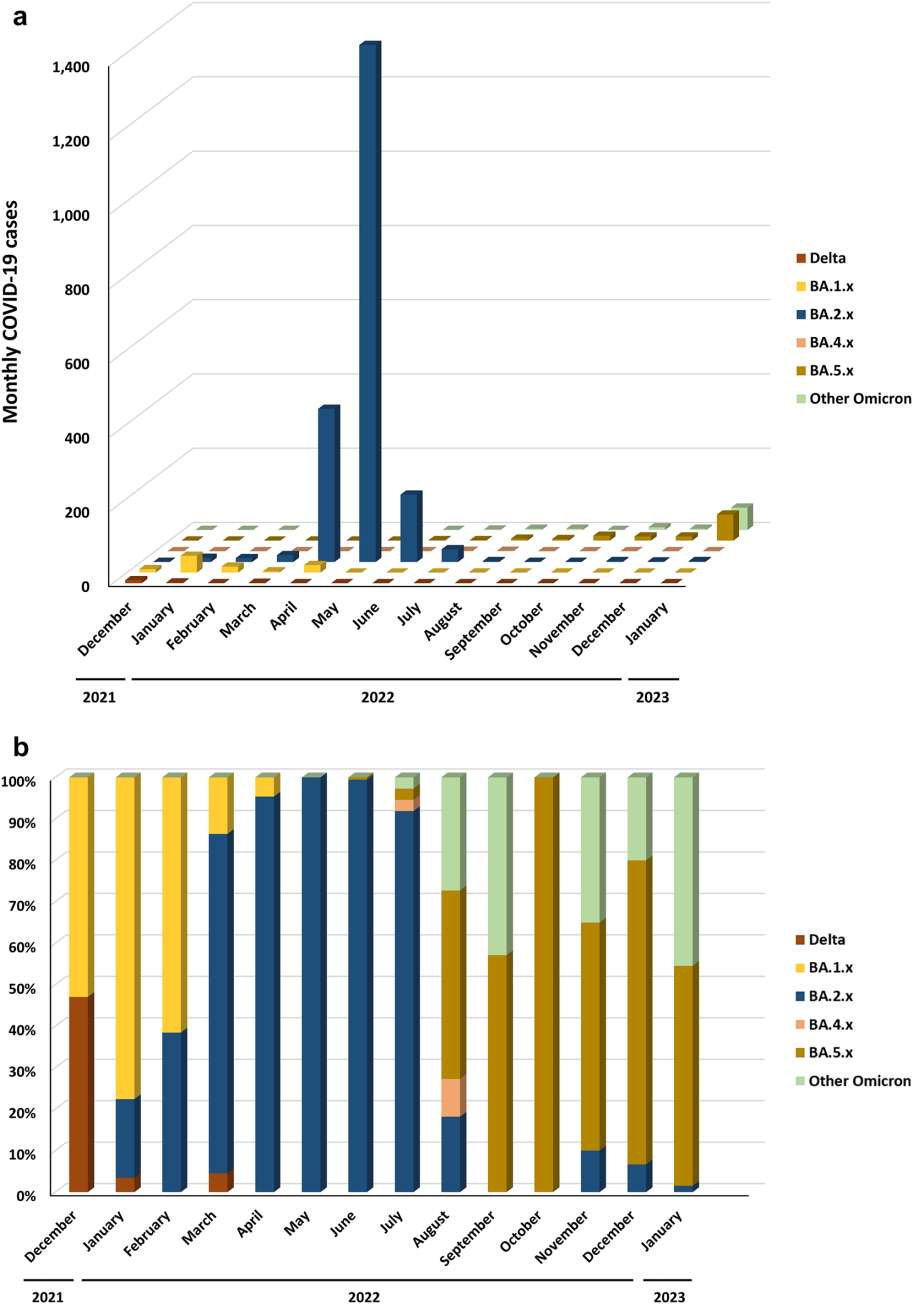
Monthly distribution of different variants of concern and clade replacement over time between December 2021 and January 2023 in Taiwan. (**a**) Distribution of the Delta variant, Omicron variant, and their subvariants. (**b**) Clade replacement during the emergence of the Omicron variant and its subvariants in Taiwan. Source of data: GISAID https://gisaid.org/.

**Figure 2:**
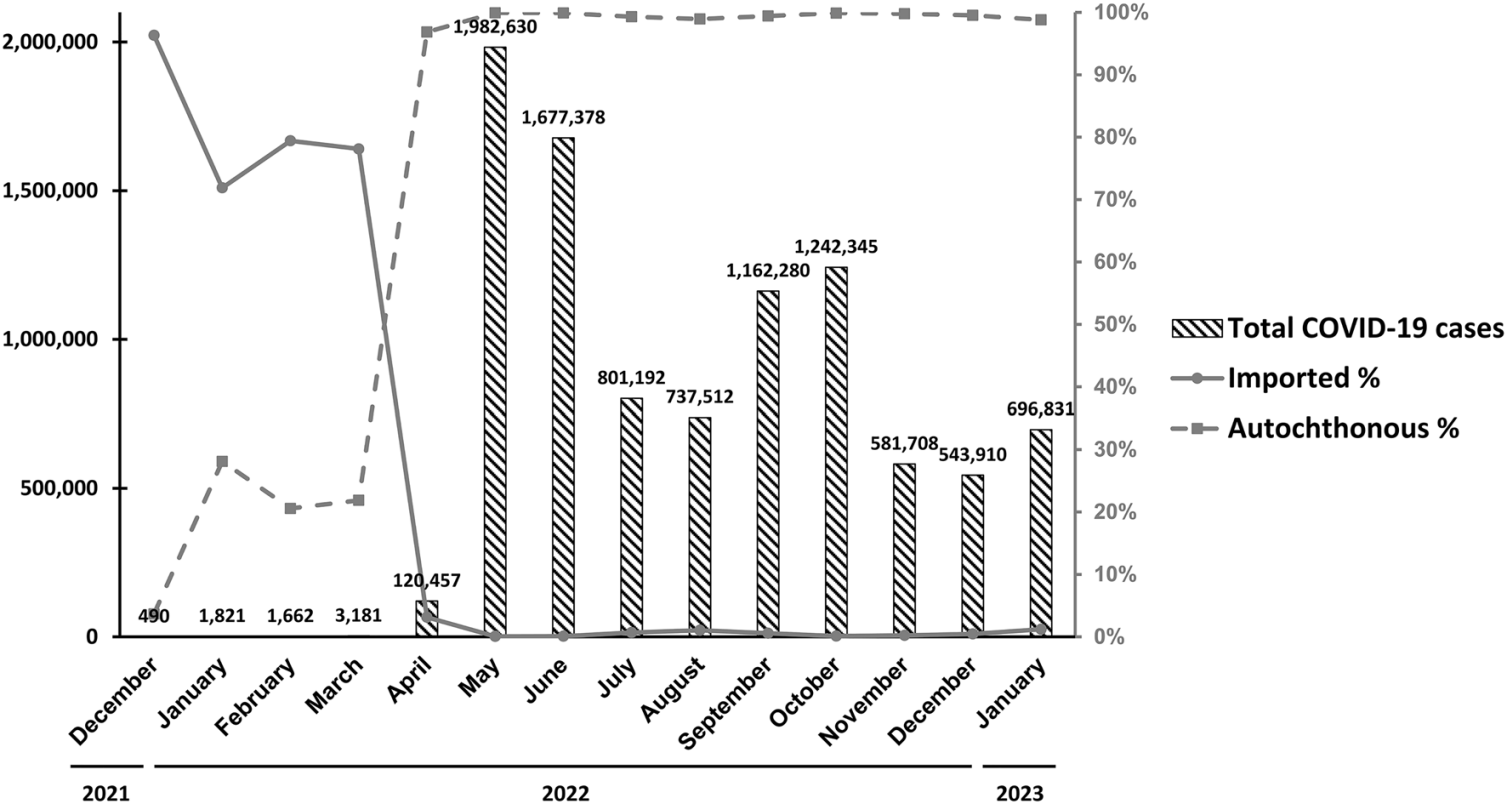
Monthly COVID-19 data between December 2021 and January 2023 in Taiwan. Source of data: The web-based notifiable disease surveillance system maintained by the Taiwan Centers for Disease Control (CDC) https://nidss.cdc.gov.tw/nndss/disease?id=19CoV.

BA.1 and BA.2 and their sublineages entered Taiwan in December 2021 and January 2022, respectively (Supplementary Table S1 and Fig. 1). These two Omicron lineages did not cause COVID-19 outbreaks until March and April 2022 (Supplementary Table S1 and Fig. 2). Most COVID-19 cases were imported from abroad in the nonoutbreak period, and autochthonous COVID-19 cases began to outnumber imported COVID-19 cases in April 2022. BA.1 lineages dominated in January and February, and BA.2 lineages gradually replaced BA.1 lineages beginning in February (Fig. 1b). COVID-19 cases correlated with BA.1 lineages were reported primarily in northern Taiwan, and BA.2 lineages spread widely in southern Taiwan. BA.2 lineages dominated between March and June, as they did worldwide. Further results revealed that BA.2.3.7 dominated between April and July 2022. BA.2.3.7 accounted for 78.7%, 86.1%, 86.8%, and 89.2% of all isolated SARS-CoV-2 infections between April and July. BA.5 and BA.4 lineages entered Taiwan in June and July 2022, respectively. BA.5 lineages replaced the BA.2 lineages rapidly and composed 54.5% to 100% of sequenced SAR-CoV-2 between August and October 2022. BA.5 lineages were gradually replaced with other Omicron lineages (e.g., BF.7, BN.1, BQ.1, and some other non-BA.5 lineages) from November 2022 to January 2023 (Fig. 1b). The geographical distribution of autochthonous cases between December 2021 and January 2023 is shown in Supplementary Fig. S1. Most of the cases were distributed in New Taipei City, Taichung City, and Kaohsiung City, which are the top three cities in terms of total population, and Taoyuan City, where the largest international airport is located. Although the sample sizes of sequences deposited in GISAID were small between August 2022 and January 2023, the trend of sequence replacement was similar to the data released by the Central Epidemic Command Center (CECC) in Taiwan. The Taiwan CECC started to release sequencing results weekly from mid-October 2022. BA.5 lineages overwhelmingly dominated between October and December 2022 (82–99%). BA.5 lineages made up 62–55% of cases during most of January 2023, declined to 35% in the last week of January and were replaced by the BA.2.75 lineage^14^.

### Detection and analysis of SARS-CoV-2 identified in this study

We collected nasopharyngeal swabs from the patients suspected with COVID-19 in KMUH from January 2022 to September 2022. Among the collected nasopharyngeal swabs, SARS-CoV-2 genomic RNA was detected in ninety-one swab-VTM samples by using real-time RT‒PCR. We evaluated the integrity of the SARS-CoV-2 genome in these swab-VTM by using virus culture. We observed a CPE in 42 out of 91 swab-VTM samples inoculated into Vero E6 cells either in cells inoculated with original swab-VTM or in cells with blind passage. We noticed that the CPE was observed only in the swab-VTM samples with a cycle threshold (Ct) value between 16.3 and 23. The presence of SARS-CoV-2 in the culture medium was confirmed by using real-time RT-PCR. The results suggested all CPEs observed were caused by the presence of SAR-CoV-2 in the culture medium. The swab-VTM samples that caused Vero E6 cells to produce a CPE were sent for next-generation sequencing for either the full-length genome or *spike* gene. The lineage and clade assignments of the sequencing results were analyzed by using the Pangolin COVID-19 Lineage Assigner^16^ and Nextclade^17^. The results revealed that 2 sequences were assigned to BA.1 lineages, 36 sequences were assigned to BA.2 lineages, and 4 sequences were assigned to BA.5 lineages. These results were basically consistent with those internationally at that time. We focused on the BA.1 lineages and BA.2 lineages in the later analysis. These sequences were uploaded to GenBank and GISAID; the demographic characteristics are listed in Supplementary Table S2. No patients died from Omicron variant infection in our study.

**Figure 3:**
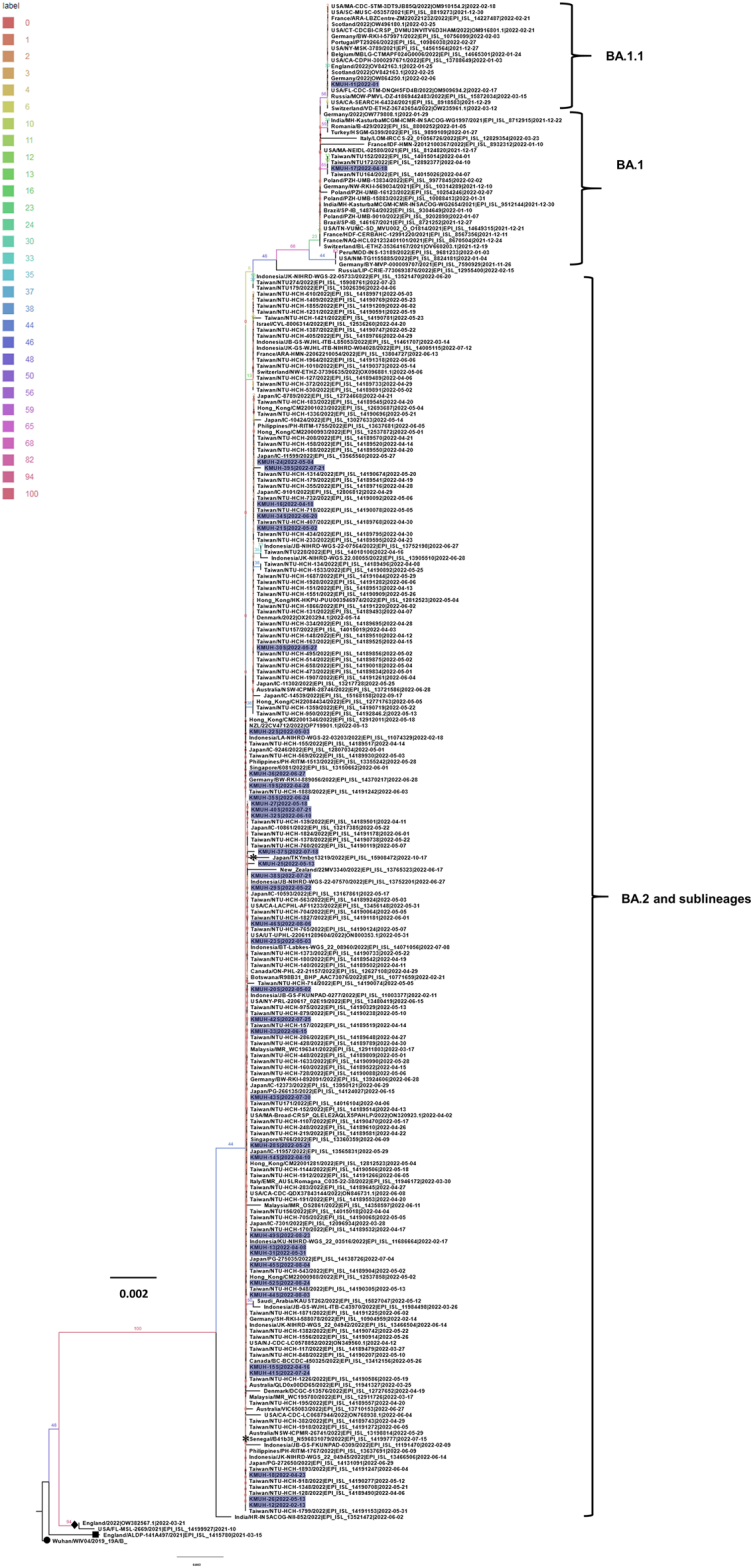
Phylogenetic analysis of Omicron subvariants identified in this study. UShER enables real-time sequence placement for the SARS-CoV-2 pandemic using an existing phylogenetic tree generated by the sarscov2phylo pipeline, containing 14,359,598 genomes from GISAID, GenBank, COG-UK, and CNCB (March 31, 2023). Some nearest neighbor SARS-CoV-2 sequences already in the existing phylogenetic tree were selected for further analysis. A total of 251 Omicron BA.1/BA.2 *spike* gene sequences, including those selected from the UShER results and *spike* gene sequences identified in this study, were analyzed by using the maximum likelihood and fits of 484 different nucleotide substitution models. The results suggested that K3Pu + F + R2 was the best-fitting model with the lowest Bayesian information criterion score of 11,355.955 among the models tested. Tree topology was automatically computed to estimate maximum likelihood values. The optimal log-likelihood for this computation was − 5089.245. There was a total of 3,180 positions in the final dataset. An original tree is displayed using FigTree v1.4.4 with a color indicator for bootstrap values and a scale bar. Viruses are shown as the Virus name| EPI_ISL ID in GISAID. The BA.1 lineage includes BA.1, BA.1.1, and BA.1.14. The BA.2 lineage includes BA.2, BA.2.3.7, BA.2.12.1, BA.2.9, and BA.2.64. The Omicron subvariants identified in this study are highlighted in purple (38 strains). Asterisk, BA.5.2; filled diamond, B.1.617.2.43 (Delta); filled square, B.1.1.7 (Alpha); filled circle, Wild-type. Wuhan/WIV04/2019 was used as the root of this tree.

To understand the phylogenetic relation of the SARS-CoV-2 variants identified in this study with those in public SARS-CoV-2 databases, such as GISAID, GenBank, COVID-19 Genomics UK Consortium, and the China National Center for Bioinformation, we analyzed the 13 full-length sequences identified in this study with Ultrafast Sample placement on Existing tRee (UShER, https://genome.ucsc.edu/cgi-bin/hgPhyloPlace) first. The analysis results suggested that the 13 full-length genomes identified in this study, including those for the BA.1, BA.1.1, BA.2, BA.2.3.7, and BA.2.64 lineages, were clustered in 11 subtrees in the existing tree with 14,359,598 SAR-CoV-2 genomes. The other 25 *spike* gene sequences were merged with selected sequences from 11 subtrees to reconstruct a single phylogenetic tree by using IQ-TREE 2.2.0 with their *spike* genes as described in our previous studies^12,15^. There were 257 *spike* sequences, including 251 Omicron BA.1/BA.2 sequences and 6 reference sequences (1 wild type, 1 Alpha variant, 2 Delta variants, and 2 Omicron BA.5.2), in the reconstructed phylogenetic tree. The results revealed that approximately 60% of BA.1, BA.2, and their sublineages in this study were located among sequences identified in Taiwan, and some others were closer to the sequences identified in other countries (Fig. 3). In general, autochthonous COVID-19 cases in Taiwan were possibly initiated by the importation of SARS-CoV-2 by travelers returning to Taiwan or international travelers visiting Taiwan.

**Figure 4:**
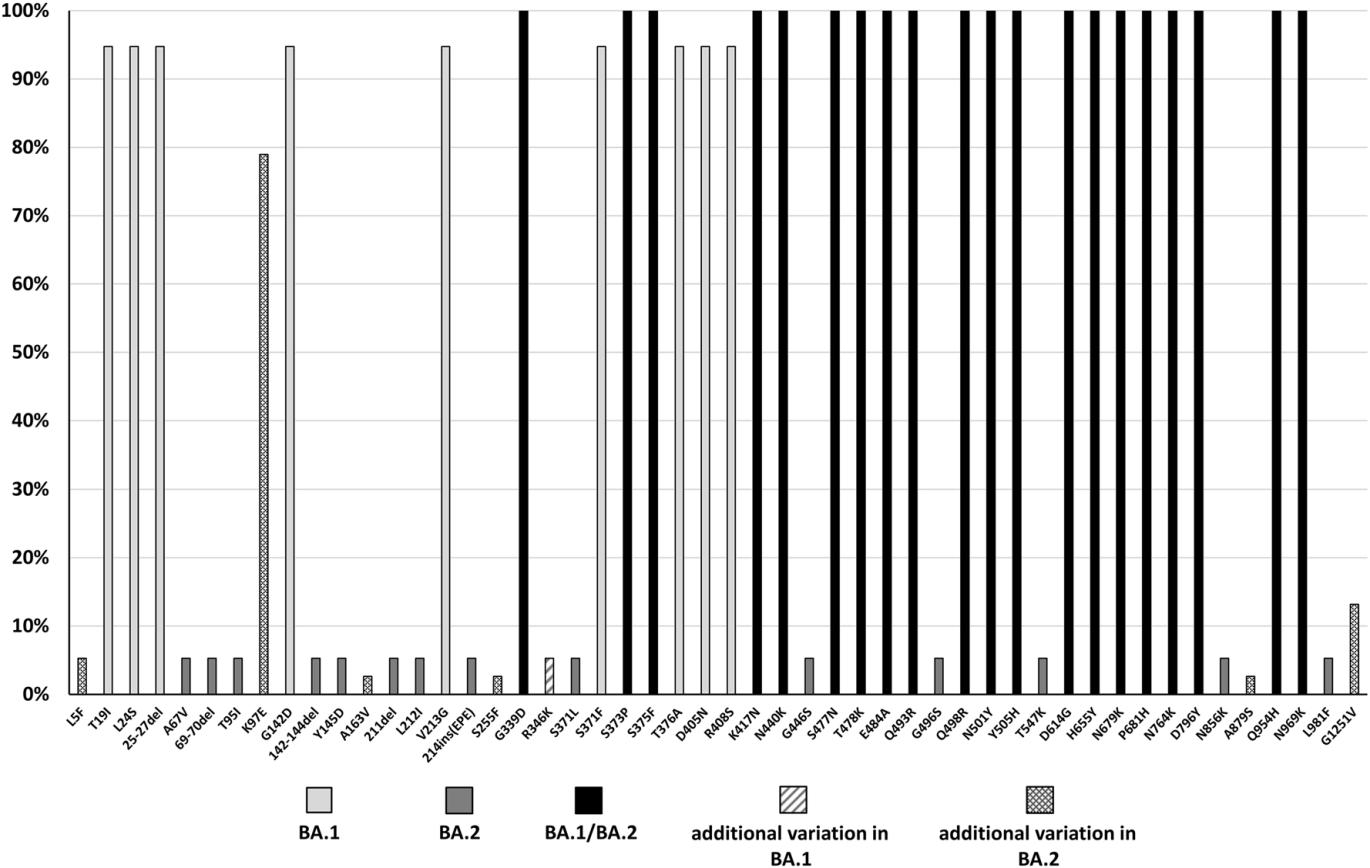
Genetic variation frequencies in BA.1, BA.2, and their sublineages isolated in this study.

We analyzed genomic variations, including single nucleotide variation (SNV) and insertion/deletion (Indel), of the 38 SARS-CoV-2 BA1 and BA.2 lineages identified in this study by comparing their sequences to the reference sequence Wuhan-Hu-1/2019 (MN908947), and we focused on the *spike* gene. The results revealed that the BA.1 and BA.2 lineages acquired additional genetic variations in the *spike* gene as they spread and evolved when compared with the original BA.1 and BA.2 lineages. These genetic variations resulted in nonsynonymous codon variations, such as L5F (5.3%), A163V (2.6%), S255F (2.6%), R346K (5.3%), A879S (2.6%), G1251V (13.2%), and K97E (78.9%) (Fig. 4). We found that spike R346K appeared only in BA.1.1, and spike K97E and G1251V were characteristic in BA.2.3.7. In addition, some reverse mutations were observed.

### *Spike* gene variations in BA.1 lineages and BA.2 lineages identified in Taiwan

We downloaded full-length genomes of BA.1 lineages and BA.2 lineages identified between December 2021 and January 2023 in Taiwan from GISAID. The quality of SARS-CoV-2 genomes was analyzed by using Nextclade, and only high-quality sequences were preserved for further analysis. The *spike* gene in 82 out of 93 BA.1 lineages, including BA.1.1, B.1.15, BA.1.16, BA.1.17, and BA.1.20 sublineages, and in 2010 out of 2069 BA.2 lineages, including BA.2.10, BA.2.3, BA.2.3.7, BA.2.68, and BA.2.75.2 sublineages, were aligned and compared with the *spike* gene of the reference sequence Wuhan-Hu-1/2019 (MN908947). The genetic Indels and SNVs resulting in amino acid (AA) deletions and nonsynonymous codon variations are shown in Table 1. The results revealed that the BA.1 sublineages and BA.2 sublineages acquired additional gene deletions and nonsynonymous codon variations in the *spike* gene compared with the original BA.1 and BA.2 lineages. Additional AA deletion in the spike protein was found at a low frequency in the BA.1 and BA.2 lineages (0.05% to 2.4%). We searched the genetic deletions found in this study in GISAID. We found 215 Omicron sequences with spike 61-73del (82.8% were in BA.1 sublineages) and 336 Omicron sequences with spike 81-95del (24.5% were in BA.1 sublineages). There was no spike 81-95del/166-180del and only 33 Omicron sequences with spike 361-376del/380-397del (3.3% were in BA.1 lineages, 36.4% were in BA.2 sublineages, and 15.2% were in BA.5.2 lineages) in the database. There were 45 Omicron sequences (including BA.1, BA.2, and BA.5 lineages) with similar spike AA deletions found in BA.2 lineages in this study. These genetic events were rare since the numbers of Omicron sequences deposited in GISAID were over 7.9 million at the time of analysis. In the context of nonsynonymous codon variations, the results revealed that additional spike R346K (40.2%) was found exclusively in BA.1 sublineages, and additional spike K97E (84.8%) and G1251V (59.5%) were found exclusively in BA.2 sublineages. Further analysis revealed that spike R346K was exclusively found in the BA.1.1 sublineage (100%). In addition, spike K97E (100%) and G1251V (71%) were characteristic mutations observed in the BA.2.3.7 sublineage (Table 2).

**Table 1:** Amino acid variations and frequencies in the spike protein of BA.1, BA.2, and their sublineages isolated and sequenced between December 2021 and January 2023 in Taiwan.

Variation type	BA.1 and its sublineages	BA.2 and its sublineages
Deletion	**69-70del** (81)	**25-27del** (2007)
	**142-144del** (81)	447-455 (1)
	**211del** (70)	447-499 (1)
	61-73del (1)	447-519 (1)
	81-95del (2)	448-520 (1)
	81-95del+166-180del (1)	449-494 (1)
	81-95del+166-180del+496-507del (1)	
	361-376del+380-397del (1)	
Insertion	**ins214EPE** (79)	
Codon change	**A67V** (72), **T95I** (76), T95V (2), **Y145D** (82), E154K (1), **L212I** (70), **G339D** (82), R346K (33), **S371L** (81), **S373P** (81), **S375F** (81), F377Y (1), **K417N** (77), **N440K** (81), **G446S** (82), **S477N** (82), **T478K** (82), **E484A** (82), **Q493R** (81), S494P (1), **G496S** (82), **Q498R** (81), **N501Y** (81), **Y505H** (81), Y508D (1), **T547K** (82), **D614G** (82), V615I (1), **H655Y** (82), **N679K** (82), **P681H** (82), R682Q (1), R682W (1), T732I (1), **N764K** (82), **D796Y** (82), **N856K** (82), **Q954H** (82), **N969K** (82), **L981F** (82)	F2L (1), L5F (9), V6F (1), **T19I** (2007), R21T (2), **L24S** (2007), W64R (20), N74D (1), T76S (1), L84I (1), K97E (1705), **G142D** (1928), H146Y (1), K147E (1), N148D (1), S151R (1), W152R (1), M153I (1), F157L (1), A163V (12), N164K (1), M177T (2), D178N (1), K182Q (1), P209T (1), I210V (1), **V213G** (2010), R214H (1), D215Y (2), A222V (1), H245N (1), L249S (4), T250P (1), S255F (1), S256L (3), G257D (1), G257S (1), P272L (2), T323I (1), **G339D** (2009), G339H (1), V341I (1), R346T (1), K356T (1), **S371F** (2010), **S373P** (2010), **S375F** (2010), **T376A** (2010), **D405N** (2010), **R408S** (1927), **K417N** (2009), **N440K** (1629), K444R (1), G446S (1), Y449H (1), N450D (1), L452M (2), N460K (2), **S477N** (2006), **T478K** (2006), **E484A** (2005), E484R (1), F490S (1), F490Y (1), **Q493R** (2002), G496S (1), **Q498R** (2003), P499T (1), **N501Y** (2004), **Y505H** (2003), K529N (1), V534F (2), T573I (1), E583D (1), **D614G** (2010), A623V (1), I651V (1), **H655Y** (2010), Q675H (1), **N679K** (2009), **P681H** (2009), A688V (3), S691P (1), N703T (3), N703Y (2), S704L (3), **N764K** (2010), R765S (1), K795R (1), **D796Y** (2010), A831V (2), R847T (1), A879S (1), A892V (1), G932V (1), L938F (2), **Q954H** (2010), **N969K** (2001), R983L (1), S1003I (1), T1009N (2), V1068I (1), G1085A (1), T1117I (1), D1139H (2), S1147L (2), K1149N (1), S1170Y (1), L1197F (3), I1225V (1), C1248R (1), G1251V (1196), S1252Y (1), P1263L (1), T1273I (1)

Some SARS-CoV-2 sequences were excluded from the analysis due to poor quality of sequencing results or the number in each sublineage (≤ 1) or the percentage (< 0.15%) being low in BA.1, BA.2, and their sublineages. This table presents data for 98.9% of BA.1 lineages (e.g., BA.1 45.2%, BA.1.1 40.9%, BA.1.15 7.5%, BA.1.16, BA.1.17, and BA.1.20; n = 82) and 99.2% of BA.2 lineages (e.g., BA.2 7.8%, BA.2.10, BA.2.3 6.2%, BA.2.3.7 84.2%, BA.2.68, and BA.2.75.2; n = 2010) identified in Taiwan in that period. Characteristic amino acid variations in BA.1 or BA.2 are labeled in bold type. Additional variations compared with the original BA.1 and BA.2 lineages are underlined. Numbers in parentheses represent the number of sequences containing the AA substitution.

**Table 2:**
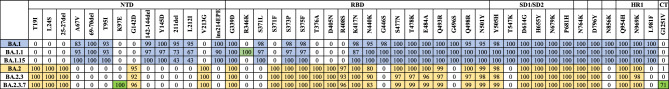
Nonsynonymous codon variations in the spike protein in the Omicron sublineages.

NTD, N-terminal domain; RBD, receptor-binding domain; SD1/SD2, subdomain 1/subdomain 2; HR1, heptad repeat 1; CT, cytoplasmic tail.

This table presents the sublineages > 5% in either the BA.1 or BA.2 lineages identified in Taiwan. The blue boxes indicate the characteristic mutations in BA.1. The yellow boxes indicate the characteristic mutations in BA.2. The green boxes indicate the characteristic mutations in either BA.1.1 or BA.2.3.7. The numbers in the table present the percentage of the characteristic AA variations in each sublineage.

### The CFR and severity of COVID-19 with Omicron subvariants

The accumulated CFR gradually decreased after BA.1 and BA.2 entered Taiwan in late 2021 and early 2022 with the accumulation of confirmed COVID-19 cases with a limited number of deaths between December 2021 and February 2022. During the outbreak related to the BA.2 sublineages, the accumulated CFR decreased drastically (Supplementary Table S1, Fig. 1, and Supplementary Fig. S2). The accumulated CFR dropped drastically after Omicron subvariants, mainly BA.2.3.7, entered Taiwan after April 2022. The CFR was 0.17% in Taiwan, which was lower than that worldwide (0.25%) between April 2022 and January 2023, and the Omicron subvariants, regardless of sublineage, dominated.

Both the accumulated and monthly CFR in Taiwan dropped rapidly after the outbreak in April 2022 to a level lower than the global CFR (Table 3). The CFRs for BA.2.x were 84.6% (monthly CFR 0.35%) and 91.7% (monthly CFR 0.31%) in April 2022 and May 2022, respectively. The dominant subvariants in Taiwan were BA.2.3.7 (86.1%, monthly CFR 0.21%) and BA.2.x (86.8%, monthly CFR 0.22%) in May 2022 and June 2022, respectively. The CFR for BA.2.x was 1.4–1.7-fold greater worldwide than that of BA.2.3.7 in Taiwan.

**Table 3:** Comparison of the global and Taiwan’s COVID-19 case fatality rates.

Year	Month	Global^a^		Taiwan^b^	
		Monthly (%)	Accumulated (%)	Monthly(%)	Accumulated(%)
2021	December	0.88	1.90	0.20	4.97
2022	January	0.27	1.51	0.11	4.50
	February	0.48	1.37	0.00	4.14
	March	0.37	1.27	0.00	3.59
	April	0.35	1.22	0.10	0.67
	May	0.31	1.19	0.18	0.21
	June	0.25	1.16	0.22	0.22
	July	0.22	1.11	0.17	0.21
	August	0.29	1.08	0.11	0.19
	September	0.34	1.06	0.13	0.18
	October	0.38	1.05	0.16	0.18
	November	0.38	1.04	0.17	0.18
	December	0.08	0.92	0.18	0.18
2023	January	0.55	0.91	0.19	0.18

^a^Source of data: Our World in Data https://covid.ourworldindata.org/data/owid-covid-data.xlsx.

^b^Source of data: Taiwan CDC https://nidss.cdc.gov.tw/nndss/disease?id=19CoV.

The COVID-19 vaccination program started in late March 2021. National vaccination rates rose rapidly in response to the COVID-19 outbreak related to the Alpha variant beginning in May 2021. The approved COVID-19 vaccines in Taiwan are AstraZeneca ChAdOx1-S (March 2021), Moderna mRNA-1273 (June 2021), BioNTech BNT162b2 (August 2021), Spikevax Bivalent Original/Omicron BA.1 (September 2022), and Spikevax Bivalent Original and Omicron BA.4/BA.5 (September 2022). Basically, the CFR was inversely correlated with the overall vaccination rate from then on (Supplementary Fig. S1).

**Figure 5:**
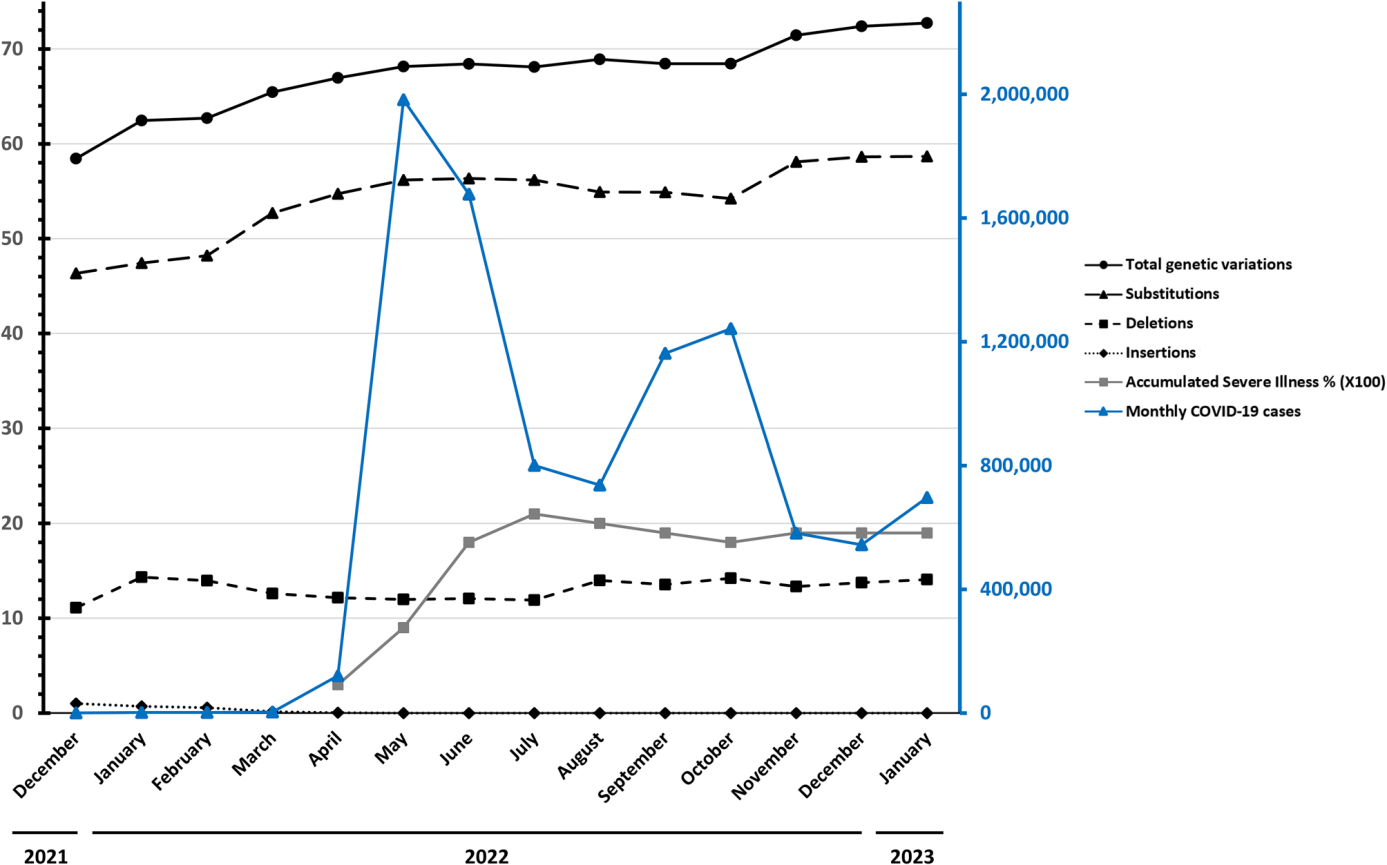
Monthly data on genetic variation frequencies in the spike protein, illness, and COVID-19 cases between December 2021 and January 2023 in Taiwan. Genetic variation frequencies are presented as average events per SARS-CoV-2 genome. The percentage of illness is the original data multiplied by 100 for presentation. Data source: GISAID https://gisaid.org/ and Taiwan CDC https://nidss.cdc.gov.tw/nndss/disease?id=19CoV.

According to the data released by the Taiwan CECC, moderate and severe COVID-19 cases increased rapidly from April 2022 after the outbreak related to BA.2 sublineages. COVID-19 patients over 70 years of age were at higher risk for moderate to severe disease and worsened after the Omicron-related outbreak, and cases increased from 60.8% in April 2022 to 71.6% in July 2022 (Supplementary Table S3). Among moderate and severe COVID-19 patients, 91% of patients had at least one chronic disease, and 91% of patients were > 60 years old. The rates for moderate and severe COVID-19 were 0.20–0.27% and 0.03–0.21%, respectively, between January 2022 and January 2023. We observed an upward trend in severe COVID-19 rates between April 2022 and July 2022 (Fig. 5). We also noted that the nonsynonymous codon variations in the spike protein resulted from genetic mutation increased during the prevalence of Omicron subvariants in Taiwan. The results suggested that AA variations might play a role in disease severity. Taken these results together, the severity of Omicron-related COVID-19 was associated with older age, the presence of chronic diseases, and the accumulation of AA variations in the spike protein.

## Discussion

Omicron was first detected in October 2021 and possess many mutations when compared to the original SARS-CoV-2. In general, the Omicron variant has increased transmissibility, lower severity, lower hospitalization rate, and lower CFR when compared to other variants (e. g. Alpha and Delta variants)^7,12,13^. The CFR of Omicron BA.1/BA.2 lineages was lower than that of the Alpha and Delta variants (0.37% between December 2021 and January 2023 vs 2.15% and 1.4–1.7%) on a global scale^12,13^. Before the Omicron variant entered Taiwan, the accumulated CFR of COVID-19 was 5.1% to 5.6% between July 2021 and November 2021 due to an outbreak related to the Alpha variants between May 2021 and June 2021. After the outbreak related to Alpha variants, the Delta variant did not cause another COVID-19 outbreak, the reasons for which were discussed in our recent study^13^. During the Omicron era of the COVID-19 pandemic, we isolated 2 BA.1 and 36 BA.2 lineages using nasopharyngeal swabs collected between January 2022 and September 2022. The isolation rate for Omicron subvariants was 46.2%, which was higher than that for wild-type SARS-CoV-2 (22.2%), Alpha variants (30.8%), and Delta variants (30%)^12,13,15^. These results agreed that the infectivity of different VOCs was generally ordered as follows: Omicron > Delta > Alpha > original SARS-CoV-2^21^. We found many additional genetic variations in the *spike* gene in the Omicron subvariants identified in this study compared with the original BA.1 and BA.2 subvariants, which were predicted to result in nonsynonymous codon variations in the spike protein, such as L5F, K97E, A163V, S255F, R346K, A879S, and G1251V. We also identified additional genetic variations resulting in nonsynonymous codon variations and AA deletions in the spike protein in the Omicron subvariants identified and sequenced in Taiwan. Our results revealed that BA.1.1 has an additional spike R346K in the receptor-binding domain (RBD) compared with BA.1. BA.1 and its derivative BA.1.1 replaced the Delta variant and rapidly became dominant between November and December 2021. Spike R346K was previously identified in the Mu variant (B.1.621) as a characteristic mutation^22^. Recent results showed that BA.1.1 harboring the spike R346K exhibited better immune escape ability than BA.1 in neutralization assays^23,24^. This result indicated that R346K caused an antigenic change in the spike protein. Spike K97E and G1251V were characteristic mutations found in BA.2.3.7. This SARS-CoV-2 subvariant was first identified in Malaysia in February 2022. BA.2.3.7 was first detected in an international traveler in Taiwan in early March 2022 and was identified as a major lineage within community outbreaks in Taiwan from April 2022 to August 2022, according to the information from GISAID and our results. This variant had a substantial cumulative prevalence in many Asia–Pacific countries during that period^25^. A recent study by Chen et al., suggested that spike K97E in the N-terminal domain (NTD) in BA.2.3.7 might contribute to the sudden increase in the incidence of extremely adverse neurological symptoms in pediatric patients. They concluded that “these severe neurological symptoms might be related to hyperimmune states rather than a direct viral invasion of the central nervous system”^26^. However, the relevance of spike K97E in BA.2.3.7 in the immune response and critical pediatric neurological illness needs to be investigated further. Although K97E was identified in B.1.428 (hCoV-19/Denmark/ALAB-HH-240/2020) in April 2020, the function of this AA substitution has not been investigated and reported in the literature. Additionally, spike G1251V, in the cytoplasmic tail (CT), was first found in B.1.1.406 (hCoV-19/Spain/AS-HUCA-232023718/2020) in March 2020. A recent molecular structure prediction study suggested that G1251V favors the formation of six additional sheets from position 1448 to 1253 and a helix at site 1254, and these alterations lie in the S2 subunit of the S protein. They suggested that spike G1251V might alter the way the spike gene interacts with the receptor to increase the infectivity of the virus to humans^27^. Their results might explain why BA.2.3.7 rapidly replaced BA.1 and BA.1.1 from April 2022 to September 2022 until BA.5.1 entered Taiwan in August 2022 and dominated in October 2022. In the phylogenetic analysis, the results suggested that approximately 60% of the sequences were more likely from autochthonous cases, while others were more similar to the sequences identified in other countries.

Taiwan had a zero-COVID strategy before March 2022. Once BA.2 dominated and the largest COVID-19 outbreak occurred, the strategy changed to coexistence with the virus in March 2022. BA.5.x and other omicron sublineages became dominated after August 2022. In April 2022, the Taiwan CECC introduced guidelines for the home care management of individuals with COVID-19. Home quarantine was recommended for individuals who test positive for COVID-19, are aged ≤ 65 years, exhibit either no symptoms or mild symptoms, and are not pregnant or undergoing kidney dialysis. The prescription and delivery of medication for confirmed individuals receiving home care followed certain principles^28^. These include the development of various telemedicine options, enabling remote consultations between patients and doctors, remote prescribing, and facilitating the dispensing and home delivery of medication by pharmacists. The most important was the introduction of effective antiviral drugs (Paxlovid and molnupiravir) for the treatment of mild-to-moderate COVID-19 and for people who are at high risk for progression to severe COVID-19, including hospitalization or death^29,30^. In September, the CECC suggested that administration of the compound monoclonal antibody tixagevimab plus cilgavimab (Evusheld) before exposure can prevent SARS-CoV-2 infection in immunocompromised populations^28^.

We observed that the accumulated CFR was inversely correlated with the overall vaccination rate (Supplementary Fig. S3). The results of a very recent multicenter study demonstrated that a mix-and-match vaccination strategy may be associated with different levels of risk reduction of infections in healthcare workers during the predominance of the SARS-CoV-2 Omicron variant in Taiwan^31^. In addition, data released by the Taiwan CECC revealed that 66% of patients who died of COVID-19 had received < 3 doses of a COVID-19 vaccine. These reports implied that a lower case fatality rate could not be attributed only to the replacement of SARS-CoV-2 variants or lineages but was likely related to increasing vaccination rates. The declining CFR was also likely correlated with the introduction of effective antiviral drugs. However, the effectiveness of antiviral drugs has not been systematically investigated in Taiwan^32^. Recent studies suggest that COVID-19 patients infected with the Omicron variant have a lower rate of hospitalization, ICU admission, and COVID-19-related mortality than people infected with previous VOCs, with a trend showing BA.2 ≤ BA.1 < Delta < Alpha^33–35^. The CFR dramatically dropped after the COVID-19 outbreak in Taiwan began in March 2022, which might be attributed to various factors, including public health actions, effective antiviral therapies, vaccination, and the lower severity of the Omicron variant. In addition, we observed that the CFR for BA.5.x subvariants, which accounted for 100% of isolates in October 2022, was 0.16% in Taiwan. Recent studies have suggested that the CFR for BA.5 subvariants was 0.86% in Macau^18^ and 0.41% in the United States of America^19^. Further results by Takahashi et al.^20^ suggested that age‑specific CFRs for cases with BA.5 sublineages at 10‑year intervals from 50, 60, 70 and ≥ 80 years were 0.03%, 0.05%, 0.39%, and 1.81%, respectively. These results suggested that the CFR of the BA.5 subvariants in Taiwan was relatively low.

We observed that the severe COVID-19 rate was higher among older COVID-19 patients (> 70 years), and comorbidity was a risk factor for COVID-19-related mortality. In addition, we found that the accumulation of AA variations in the spike protein possibly played a role in COVID-19 severity during the Omicron epidemic between April 2022 and January 2023. Maurya et al.^36^ observed a higher number of mutations per sample in mortality cases than in the recovered group (28.94 vs. 13.60). Severity-related mutations were identified and found to be located across the SARS-CoV-2 genome. For example, spike L18F, F58L, C361C*, L452R, N501Y, D614G, and V1176F were found to be correlated with severity, although different results have also been reported^36,37^. Although Omicron displayed lower hospitalization rate, ICU admission, need of oxygenation/ventilation, and death regardless of the vaccination status or doses generally^7^, certain studies have reported a similar severity level in patients infected with the Omicron variant as with other variants, highlighting a significant risk of severe illness^8–10^. In addition to viral factors, it has been suggested that different ethnic or genetic backgrounds, immune statuses, and environmental, clinical and social factors affect mortality^38,39^. Thus, more investigations need to be conducted to generalize our observations and provide a basis for the pathogenic mechanisms in our study.

There are some limitations in this study. First, the SARS-CoV-2 sequences generated in this study and downloaded from the public SARS-CoV-2 sequence repositories may not fully represent all epidemic Omicron variants during the time investigated. Thus, the results of the phylogenetic tree reconstruction and clade replacement analysis between December 2021 and January 2023 in this study might be compromised. It is noteworthy that phylogenetic analysis only explains the distance between the sequences but does not indicate the evolutionary distance between them. Second, we did not obtain access to the clinical information of confirmed COVID-19 patients or countrywide vaccination information. Thus, we could not analyze the effectiveness of different vaccines in the prevention of SARS-CoV-2 infection and severe COVID-19. Third, we correlated accumulating AA variations only in the spike protein with the increasing severity of COVID-19 due to the Omicron variant. However, AA variations in other viral proteins might also affect the pathogenesis of SARS-CoV-2 in humans. The molecular mechanism is unknown and remains to be elucidated.

## Conclusions

In this study, we detected and identified BA.1, BA.2, and their sublineages (80.0% were BA.2.3.7) in the COVID-19 outbreak related to Omicron in Taiwan between January 2022 and September 2022. The results of sequence placement and phylogenetic tree reconstruction revealed that 60% of these Omicron strains were more likely from autochthonous cases identified in Taiwan. The SARS-CoV-2 replacement analysis using data from the GISAID suggested that BA.2.3.7 emerged in March 2022 and possibly persistently dominated the other VOCs between April 2022 and August 2022. Older patients were more likely to have severe COVID-19, and comorbidity was a risk factor for COVID-19-related mortality during the Omicron outbreak. The accumulation of AA variations in the spike protein was accompanied by increased COVID-19 severity during the Omicron epidemics between April 2022 and January 2023. However, this observation needs to be verified by further investigation. The low CFR during the Omicron epidemics in Taiwan can be attributable to rapid adjustments to public health policies, rapid promotion of vaccination programs, effective antiviral treatments and the lower severity of the Omicron variant.

## Materials and methods

### Ethics and sample collection

This study was reviewed and approved by the Institutional Review Board of Kaohsiung Medical University Hospital (KMUHIRB-E-I-20200013). Nasopharyngeal swabs were collected from patients suspected of having COVID-19 at Kaohsiung Medical University Hospital (KMUH) between January 2022 and September 2022. The swabs were maintained in Viral Transport Medium (VTM) (Creative Life Science, Taiwan).

### Detection of SARS-CoV-2 genome by real-time RT‒PCR

We detected the presence of the SARS-CoV-2 genome by using the Cobas SARS-CoV-2 test in a Cobas 6800 System (Roche Molecular Systems, Inc., NJ, USA) or by using the Cobas SARS-CoV-2 & Influenza A/B test in a Liat Analyzer (Roche Molecular Systems, Inc., NJ, USA) according to the manufactures’ instructions. Both are sample in–result out systems. The Cobas SARS-CoV-2 test detects unique sequences in the RNA-dependent RNA polymerase (*RdRP*) gene and envelope (*E*) gene of the SARS-CoV-2 genome. Cobas SARS-CoV-2 & Influenza A/B targets both the *RdRP* gene and nucleocapsid (*N*) gene that are unique to SARS-CoV-2, a well-conserved region of the matrix gene of Influenza A, and the non-structural protein gene of Influenza B. The results are interpreted automatically by each of the two systems.

### Isolation of SARS-CoV-2 in Vero E6 cells

Vero E6 cells were routinely maintained in DMEM supplemented with 1× antibiotics and 10% fetal bovine serum. Swab-VTM samples with a positive qRT–PCR result were inoculated into Vero E6 cells to isolate SARS-CoV-2 as described in our previous studies^12,15^. The cytopathic effect (CPE) was examined daily under a phase-contrast microscope. For samples that did not show a CPE after three days of incubation, a blind passage was performed until day 21 to increase the chance for virus propagation and isolation, with medium replacement every 2–3 days.

### RNA library construction and sequence analysis

Viral RNA extraction, RNA library construction, next-generation sequencing (NGS) and sequence analysis were performed using swab-VTM samples as described in our previous studies^12,15^.

### PCR amplification and Sanger sequencing

*Spike* gene fragments were amplified for Sanger sequencing by the methods described in our previous studies^12,15^. The primer pairs used are listed in Supplementary Table S4.

### Retrieval of COVID-19 and nationwide vaccination data in Taiwan

The COVID-19 data, including monthly imported and autochthonous COVID-19 cases and COVID-19-related deaths in Taiwan, were retrieved from the web-based notifiable diseases surveillance system (https://nidss.cdc.gov.tw/nndss/disease?id=19CoV) maintained by the Taiwan CDC. The data on moderate and severe COVID-19 were obtained from daily press conferences held by the Taiwan CECC (http://at.cdc.tw). Nationwide vaccination data were retrieved from the Taiwan CDC website (https://www.cdc.gov.tw/). These data are publicly available.

### SARS-CoV-2 genome and phylogenetic analysis

Genomic sequences of SARS-CoV-2 were retrieved and downloaded from the Global Initiative on Sharing all Influenza Data (GISAID) EpiCoV (https://www.gisaid.org/) and National Center for Biotechnology Information (NCBI) GenBank (https://www.ncbi.nlm.nih.gov/genbank/) databases. The SARS-CoV-2 genomic sequences were multi-aligned by using MAFFT v 7.511^40^. The best evolutionary model was investigated by using ModelFinder^41^. Theoretical phylogenetic tree was reconstructed by using IQ-TREE 2.2.0 COVID-19 edition with 1000 bootstrap replicates as described in our previous studies^12,15^.

## Supplementary Information

Supplementary Information.

## Data Availability

The datasets presented in this study can be found in online repositories. The names of the repository/repositories and accession number(s) can be found in the figures, tables, and footnotes.
