# Author Correction: Epidemiology and analysis of SARS-CoV-2 Omicron subvariants BA.1 and 2 in Taiwan

**DOI:** 10.1038/s41598-024-78547-4

**Published:** 2024-11-07

**Authors:** Li-Teh Liu, Shyh-Shin Chiou, Po-Chih Chen, Chun-Hong Chen, Ping-Chang Lin, Ching-Yi Tsai, Wan-Long Chuang, Shang-Jyh Hwang, Inn-Wen Chong, Jih-Jin Tsai

**Affiliations:** 1 Department of Medical Laboratory Science and Biotechnology, College of Medical Technology, Chung Hwa University of Medical Technology, Tainan City, Taiwan; 2 Graduate Institute of Clinical Medicine, College of Medicine, Kaohsiung Medical University, Kaohsiung City, Taiwan; 3 Center of Applied Genomics, Kaohsiung Medical University, Kaohsiung City, Taiwan; 4 Division of Pediatric Hematology and Oncology, Department of Pediatrics, Kaohsiung Medical University Hospital, Kaohsiung City, Taiwan; 5 Department of Laboratory Medicine, Kaohsiung Medical University Hospital, Kaohsiung City, Taiwan; 6 Department of Medical Laboratory Science and Biotechnology, Kaohsiung Medical University, Kaohsiung City, Taiwan; 7 National Mosquito-Borne Diseases Control Research Center, National Health Research Institutes, Zhunan, Miaoli County Taiwan; 8 National Institute of Infectious Diseases and Vaccinology, National Health Research Institutes, Zhunan, Miaoli County Taiwan; 9 Tropical Medicine Center, Kaohsiung Medical University Hospital, Kaohsiung City, Taiwan; 10 School of Medicine, College of Medicine, Kaohsiung Medical University, Kaohsiung City, Taiwan; 11 Hepatobiliary Division, Department of Internal Medicine, Kaohsiung Medical University Hospital, Kaohsiung City, Taiwan; 12 Division of Nephrology, Department of Internal Medicine, Kaohsiung Medical University Hospital, Kaohsiung City, Taiwan; 13 Department of Internal Medicine and Graduate Institute of Medicine, Kaohsiung Medical University, Kaohsiung City, Taiwan; 14 Department of Pulmonary Medicine, Kaohsiung Medical University Hospital, Kaohsiung City, Taiwan; 15 Division of Infectious Diseases, Department of Internal Medicine, Kaohsiung Medical University Hospital, No. 100, Tzyou 1st Road, Kaohsiung City, 80756 Taiwan

Correction to: *Scientific Reports* 10.1038/s41598-023-43357-7, published online 03 October 2023

The original version of this Article contained errors in Figure 5 where the legends for the genetic variation frequencies in the spike protein and the Y-axis label were incorrect. The original Figure 5 and accompanying legend appear below.

**Fig. 5:**
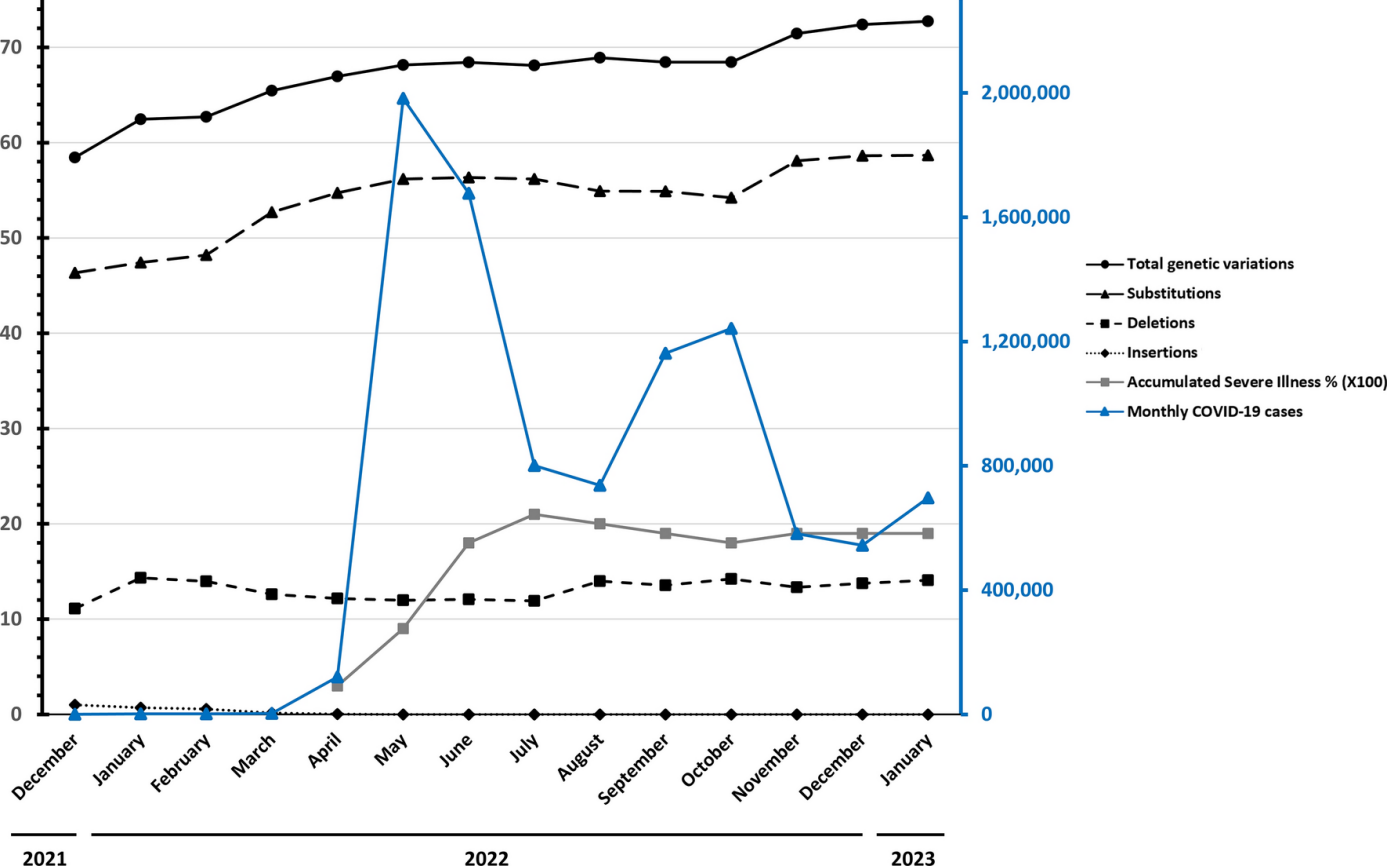
Monthly data on genetic variation frequencies in the spike protein, illness, and COVID-19 cases between December 2021 and January 2023 in Taiwan. The percentage of illness is the original data multiplied by 100 for presentation. Data source: GISAID https://gisaid.org/ and Taiwan CDC https://nidss.cdc.gov.tw/nndss/disease?id=19CoV.

In addition, in the Discussion section,

Their results might explain why BA.2.3.7 rapidly replaced BA.1 and BA.1.1 from April 2020 to September 2022 until BA.5.1 entered Taiwan in August 2022 and dominated in October 2022.

now reads:

Their results might explain why BA.2.3.7 rapidly replaced BA.1 and BA.1.1 from April 2022 to September 2022 until BA.5.1 entered Taiwan in August 2022 and dominated in October 2022.

The original Article has been corrected.

