# Supplementary material for: Epidemiology and analysis of SARS-CoV-2 Omicron subvariants BA.1 and 2 in Taiwan
Source: Sci Rep. 2023 Oct 3;13:16583. doi: 10.1038/s41598-023-43357-7 (PMC10547678; doi:10.1038/s41598-023-43357-7)
Supplement: Supplementary file 1 — Supplementary Information. [file 41598_2023_43357_MOESM1_ESM.pdf]

# Supplementary Information

**Supplementary Figure S1**. **Geographical distribution of autochthonous COVID-19 cases between December 2021 and January 2023 in Taiwan.** Confirmed COVID-19 case numbers are shown for each city in Taiwan. The numbers in the brackets are COVID-19-related deaths. This figure was generated using QGIS v3.30 (QGIS Development Team, 2023, QGIS Geographic Information System, Open Source Geospatial Foundation, http://www.qgis.org/). Taiwan map data were retrieved from the Taiwan Geospatial One-Stop Portal developed by the Information Center of the Taiwan Ministry of the Interior and used under the Open Government Data License.

**Supplementary Figure S2**. **Accumulated data on confirmed cases, deaths, and case fatality rates (CFRs) of COVID-19 between December 2021 and January 2023 in Taiwan.** Source of data: http://at.cdc.tw.

**Supplementary Figure S3**. **Accumulated data on the vaccination rate and case fatality rate (CFR) of COVID-19 between December 2021 and January 2023 in Taiwan.** Data include only citizens ≥ 12 years old before April 2022. Data include citizens ≥ 5 years old between May 2022 and November 2022. Data include citizens ≥ 6 months between December 2022 and January 2023. The approved COVID-19 vaccines in Taiwan are AstraZeneca ChAdOx1-S (March 2021), Moderna mRNA-1273 (June 2021), BioNTech BNT162b2 (August 2021), Spikevax Bivalent

Original/Omicron BA.1 (September 2022), and Spikevax Bivalent Original and Omicron BA.4/BA.5 (September 2022). Source of data: http://at.cdc.tw.

**Supplementary Table S1. Countrywide COVID-19 data between December 2021 and January 2023 in Taiwan.**

**Supplementary Table S2. Information on SARS-CoV-2 subvariants identified and sequenced in this study.**

**Supplementary Table S3. Accumulated data on moderate and severe COVID-19 cases between January 2022 and January 2023 in Taiwan.**

**Supplementary Table S4. Primer sequences, annealing temperature, and amplicon length in the amplification and Sanger sequencing of the *spike* gene.**

Supplementary Figure S1

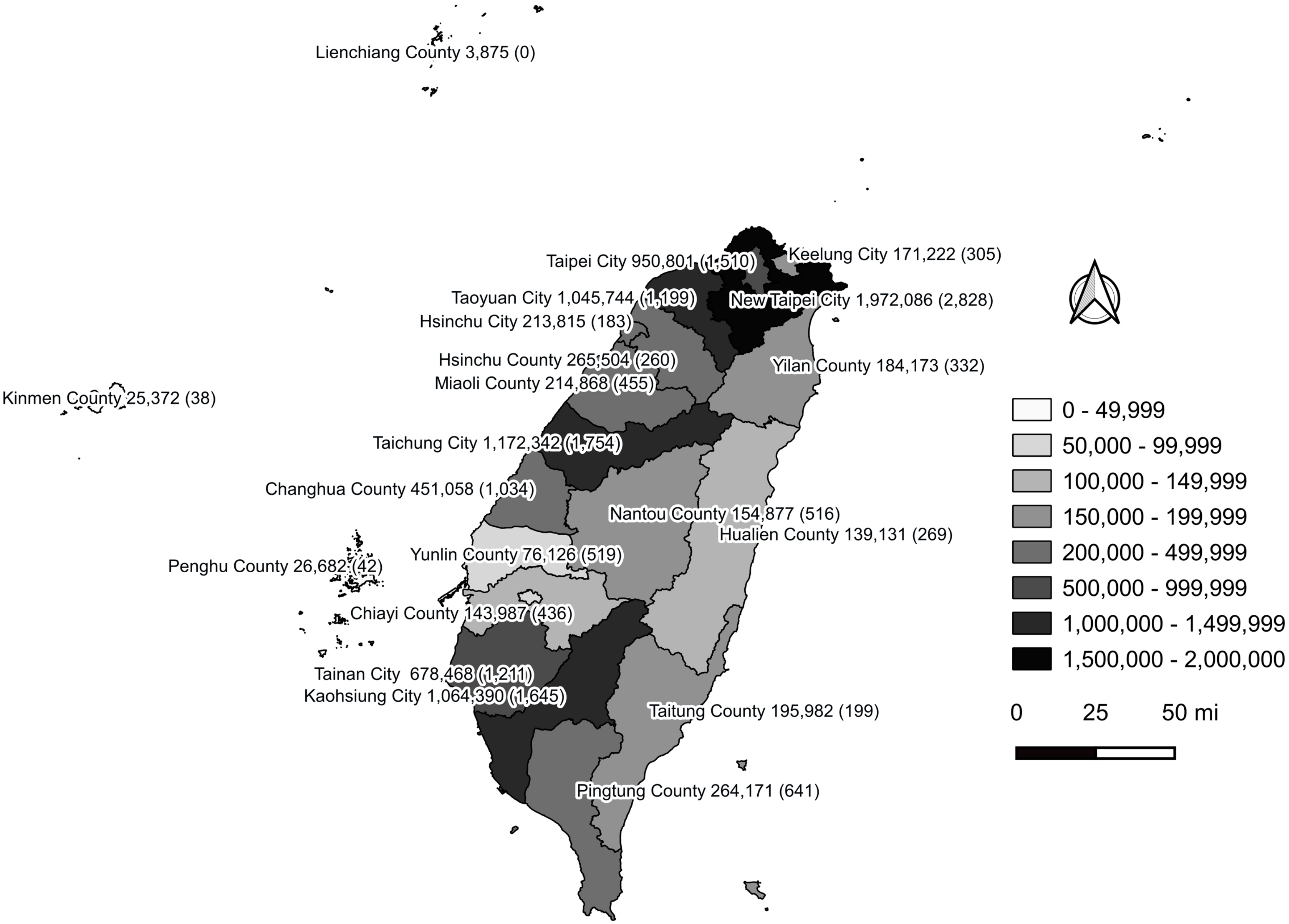

Supplementary Figure S2

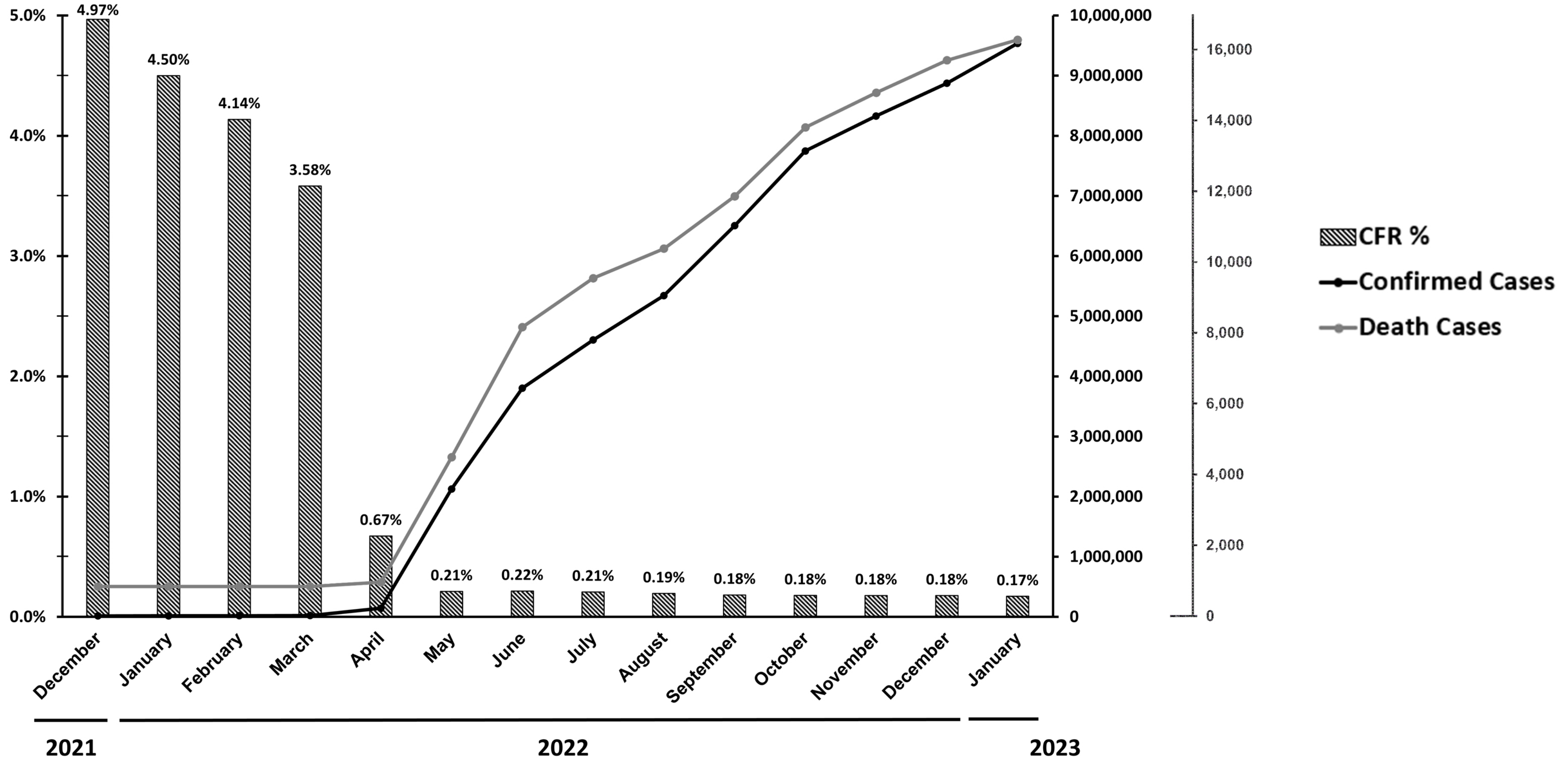

Supplementary Figure S3

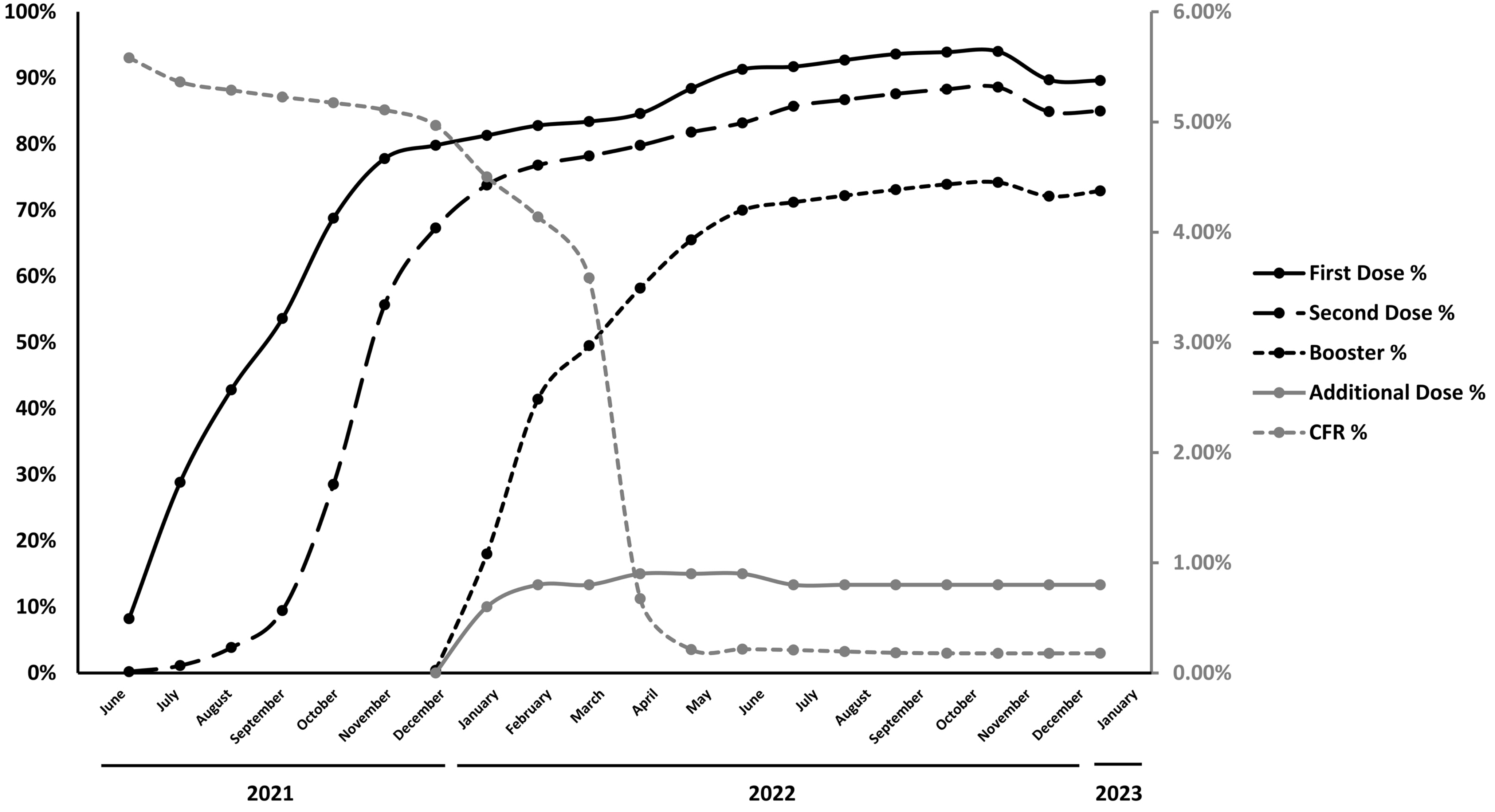

**Supplementary Table S1. Countrywide COVID-19 data between December 2021 and January 2023 in Taiwan.**[a]

| Year | Month | Autochthonous | Imported | Confirmed | Deaths | CFR[b] % | Clade/Lineage[c] | |
|---|---|---|---|---|---|---|---|---|
| | | | | | | | Dominant | Others |
| 2021 | December | 18 | 472 | 490 | 1 | 0.20% | BA.1.1 | AY.103, B.1.617.2, BA.1 |
| | January | 512 | 1,309 | 1,821 | 2 | 0.11% | BA.1.1 | BA.1, BA.1.15, BA.2.3, BA.2.10 |
| | February | 342 | 1,320 | 1,662 | 0 | 0.00% | BA.1.1, BA.1 | BA.2, BA.2.2, BA.2.3 |
| | March | 696 | 2,485 | 3,181 | 0 | 0.00% | BA.1.1, BA.2, BA.2.10, BA.2.3.7 | BA.2.3, BA.2.3.2, BA.2.1.17 |
| | April | 116,662 | 3,795 | 120,457 | 119 | 0.10% | BA.2.3.7 | BA.2, BA.2.3, BA.1 |
| | May | 1,980,998 | 1,632 | 1,982,630 | 3,542 | 0.18% | BA.2.3.7 | BA.2, BA.2.3, BA.2.68 |
| 2022 | June | 1,675,643 | 1,735 | 1,677,378 | 3,681 | 0.22% | BA.2.3.7 | BA.2.3, BA.2, BA.5.6, BA.2.68 |
| | July | 795,647 | 5,545 | 801,192 | 1,382 | 0.17% | BA.2.3.7 | BA.4.1, BA.5.2.1, BE.1.1 |
| | August | 729,732 | 7,780 | 737,512 | 836 | 0.11% | BA.2.3.7, BA.5.1 | BA.5.2.x, BF.10 |
| | September | 1,155,433 | 6,847 | 1,162,280 | 1,480 | 0.13% | BA.2.3.7, BF.10 | BA.5.2.x, BF.10 |
| | October | 1,240,832 | 1,513 | 1,242,345 | 1,954 | 0.16% | BA.5.1 | BA.5.2.x |
| | November | 580,354 | 1,354 | 581,708 | 978 | 0.17% | BA.5.1, BA.5.2.1 | BF.29, BN.1.4, CM.2, XBB.1 |
| | December | 541,368 | 2,542 | 543,910 | 953 | 0.18% | BF.x, BN.x | BA.5.1, BA.5.2, BE.1.1 |
| 2023 | January | 688,389 | 8,442 | 696,831 | 1,310 | 0.19% | BA.5.2.48 | BF.7.14, BA.5.2.1, BA.5.2 |

[a] COVID-19 data were retrieved from the web-based notifiable disease surveillance system maintained by the Taiwan CDC. Source of data: https://nidss.cdc.gov.tw/nndss/disease?id=19CoV.

[b] CFR: Accumulated case fatality rate

[c] According to the data deposited in GISAID (https://gisaid.org/).

**Supplementary Table S2. Information on SARS-CoV-2 subvariants identified and sequenced in this study.**

| ID in this study[a] | Nextclade_pango | GISAID_EPI_ISL | GenBank_Accession | Collection Date[b] | Underlying Disease | Vaccination Status[c] |
|---|---|---|---|---|---|---|
| KMUH-11 | BA.1.1 | EPI_ISL_12005452.1 | OP825137 | 2022/1/5 | No | B+B |
| KMUH-12 | BA.2.64 | EPI_ISL_15757354 | OP825138 | 2022/2/13 | No | A+A |
| KMUH-13 | BA.2.3.7 | EPI_ISL_15757448 | OP825139 | 2022/4/8 | No | A+A+M |
| KMUH-14S | BA.2.3.7 | EPI_ISL_17463783 | OP825140 | 2022/4/10 | No | M |
| KMUH-15S | BA.2.3.7 | EPI_ISL_17463884 | OP825141 | 2022/4/16 | No | A |
| KMUH-16 | BA.2.3.7 | EPI_ISL_15757747 | OP825142 | 2022/4/18 | No | M+M+M |
| KMUH-17 | BA.1 | EPI_ISL_15757893 | OP825143 | 2022/4/18 | No | A+A+M |
| KMUH-18 | BA.2.3.7 | EPI_ISL_15757893 | OP825144 | 2022/4/23 | No | G+G+G |
| KMUH-19S | BA.2 | EPI_ISL_17464048 | OP825145 | 2022/4/28 | No | A+A+B |
| KMUH-20S | BA.2.3.7 | EPI_ISL_17464049 | OP825146 | 2022/5/2 | Type 2 diabetes, Hypertension, stage 2 CKD, Rectal cancer lung metastasis | M |
| KMUH-21S | BA.2.3.7 | EPI_ISL_17464050 | OP825147 | 2022/5/2 | Type 2 diabetes, Hypertension | No |
| KMUH-22S | BA.2 | EPI_ISL_17464051 | OP825148 | 2022/5/3 | Type 2 diabetes | M+M+M |
| KMUH-23S | BA.2.3.7 | EPI_ISL_17464052 | OP825149 | 2022/5/3 | Hepatitis B carrier | No |
| KMUH-24 | BA.2.3.7 | EPI_ISL_15757895 | OP825150 | 2022/5/4 | No | M+M+M |

| | | | | | | |
|---|---|---|---|---|---|---|
| KMUH-25 | BA.2.3.7 | EPI_ISL_15757905 | OP825151 | 2022/5/13 | Type 2 diabetes, Hypertension | A+A+M |
| KMUH-26 | BA.2 | EPI_ISL_15757924 | OP825152 | 2022/5/13 | Type 2 diabetes, Hypertension, Cholelithiasis with acute cholecystitis, Communicating hydrocephalus, Dyslipidemia | A+A+M |
| KMUH-27 | BA.2.3.7 | EPI_ISL_15758299 | OP825153 | 2022/5/18 | None | A+A+M |
| KMUH-28S | BA.2.3.7 | EPI_ISL_17468450 | OP825154 | 2022/5/21 | Type 2 diabetes, Hypertension | No |
| KMUH-29S | BA.2.3.7 | EPI_ISL_17468453 | OP825155 | 2022/5/22 | End-stage renal disease | A+A+M |
| KMUH-30S | BA.2.3.7 | EPI_ISL_17468454 | OP825156 | 2022/5/27 | No | No |
| KMUH-31 | BA.2 | EPI_ISL_16178270 | OQ194009 | 2022/5/31 | No | M+M+M |
| KMUH-32S | BA.2.3.7 | EPI_ISL_17468455 | OP825157 | 2022/6/10 | Burkitt lymphoma | No |
| KMUH-33 | BA.2.3.7 | EPI_ISL_16178272 | OQ194010 | 2022/6/15 | No | A+A |
| KMUH-34S | BA.2.3.7 | EPI_ISL_17468456 | OP825158 | 2022/6/20 | No | M+M+M |
| KMUH-35S | BA.2.3.7 | EPI_ISL_17468457 | OP825159 | 2022/6/24 | No | None |
| KMUH-36 | BA.2.3 | EPI_ISL_16178276 | OQ194011 | 2022/6/27 | No | B+B |
| KMUH-37S | BA.2.3.7 | EPI_ISL_16873059 | OQ082558 | 2022/7/18 | No | A+A |

| | | | | | | |
|---|---|---|---|---|---|---|
| KMUH-38S | BA.2.3.7 | EPI_ISL_16873058 | OQ082559 | 2022/7/21 | No | B+B+M |
| KMUH-39S | BA.2.3.7 | EPI_ISL_16873057 | OQ082560 | 2022/7/21 | No | A+M |
| KMUH-40S | BA.2.3.7 | EPI_ISL_16873056 | OQ082561 | 2022/7/21 | No | No |
| KMUH-41S | BA.2.3.7 | EPI_ISL_16873055 | OQ082562 | 2022/7/24 | Type 2 diabetes, Occlusion and stenosis of the carotid artery, Diabetes mellitus, Hypertension | No |
| KMUH-42S | BA.2.3.7 | EPI_ISL_16873054 | OQ082563 | 2022/7/25 | No | No |
| KMUH-43S | BA.2.3.7 | EPI_ISL_16873053 | OQ082564 | 2022/7/30 | No | B |
| KMUH-44S | BA.2.3.7 | EPI_ISL_16873052 | OQ082565 | 2022/8/3 | No | No |
| KMUH-45S | BA.2.3.7 | EPI_ISL_16873051 | OQ082566 | 2022/8/4 | No | A+A+M+M |
| KMUH-46S | BA.2.3.7 | EPI_ISL_16873050 | OQ082567 | 2022/8/6 | No | B+B |
| KMUH-49S | BA.2.3.7 | EPI_ISL_16873049 | OQ082568 | 2022/8/8 | No | No |
| KMUH-52S | BA.2.3.7 | EPI_ISL_16873047 | OQ082569 | 2022/9/1 | No | No |

[a] An ID with a S indicates it is a *spike* gene sequence. Otherwise, it is a full-length sequence.

[b] YYYY/MM/DD

[c] A: AstraZeneca); M: Moderna); B: BNT; G: Medigen (MVC COVID-19 vaccine, made in Taiwan)

**Supplementary Table S3. Accumulated data on moderate and severe COVID-19 cases between January 2022 and January 2023 in Taiwan.**[a]

| | **April 2022** | | | **July 2022** | | | **October 2022** | | | **January 2023** | | |
|---|---|---|---|---|---|---|---|---|---|---|---|---|
| Age Group | **Moderate** | **Severe** | **%** | **Moderate** | **Severe** | **%** | **Moderate** | **Severe** | **%** | **Moderate** | **Severe** | **%** |
| 0-9 | 0 | 1 | 0.5% | 279 | 122 | 1.9% | 432 | 224 | 2.0% | 525 | 267 | 1.8% |
| 10-19 | 3 | 1 | 1.9% | 78 | 32 | 0.5% | 128 | 60 | 0.6% | 170 | 71 | 0.6% |
| 20-29 | 9 | 0 | 4.4% | 126 | 42 | 0.8% | 202 | 61 | 0.8% | 247 | 79 | 0.8% |
| 30-39 | 8 | 0 | 3.9% | 188 | 88 | 1.3% | 312 | 135 | 1.4% | 391 | 151 | 1.3% |
| 40-49 | 8 | 0 | 3.9% | 397 | 267 | 3.2% | 677 | 405 | 3.3% | 877 | 492 | 3.2% |
| 50-59 | 12 | 3 | 7.3% | 905 | 602 | 7.3% | 1483 | 924 | 7.3% | 1924 | 1145 | 7.1% |
| 60-69 | 34 | 3 | 18.0% | 1832 | 1300 | 15.2% | 3047 | 1953 | 15.3% | 3988 | 2508 | 15.1% |
| 70-79 | 34 | 3 | 18.0% | 2636 | 2045 | 22.7% | 4342 | 3047 | 22.6% | 5723 | 3912 | 22.3% |
| 80-89 | 56 | 8 | 31.1% | 3520 | 3050 | 31.9% | 5847 | 4536 | 31.7% | 7683 | 5919 | 31.5% |
| 90- | 21 | 3 | 11.7% | 1676 | 1831 | 17.0% | 2827 | 2764 | 17.1% | 3724 | 3625 | 17.0% |
| Subtotal | 185 | 21 | 100.0% | 11358 | 9257 | 100.0% | 18865 | 13885 | 100.0% | 25252 | 17902 | 100.0% |
| Total | 206 | | | 20615 | | | 32750 | | | 43154 | | |
| Death (%) | 12 (5.8%) | | | 8074 (39.2%) | | | 11976 (36.6%) | | | 15497 (35.9%)[b] | | |

[a] Source of data: http://at.cdc.tw.

[b] 91% of patients had at less one chronic disease. 66% of patients received < 3 doses of COVID-19 vaccines. 91% of patients are > 60 years old. Death percentage was calculated as a % (number of death cases/total number of moderate and severe cases). The information was released by Taiwan CECC on 1 February, 2023 and included the data between 1 January, 2022 to 31 January, 2023.

**Supplementary Table S4. Primer sequences, annealing temperature, and amplicon length in the amplification and Sanger sequencing of the *spike* gene.**

| Position | Primer Name | Primer sequence (5'-3') | Annealing (°C) | Amplicon (bp) |
|---|---|---|---|---|
| 21428-22038 | Spike-21428-F<br>Spike-22038-R | CTGCTGTTATGTCTTTAAAAGAAGGTCA<br>ACTCTGAACTCACTTTCCATCCAAC | 60 | 611 |
| 21962-22537 | Spike-21962-F<br>Spike-22537-R | CAATTTTGTAATGATCCATTTTTGGGTGT<br>AGATTCTGTTGGTTGGACTCTAAAGT | 60 | 576 |
| 22263-22903 | Spike-22263-F<br>Spike-22903-R | ACATCACTAGGTTTCAAACTTTACTTGC<br>ACCACCAACCTTAGAATCAAGATTGT | 60 | 641 |
| 22799-23426 | Spike-22799-F<br>Spike-23426-R | GGGCAAACTGGAAAGATTGCTGA<br>CAGGGACTTCTGTGCAGTTAACA | 60 | 628 |
| 23240-23847 | Spike-23240-F<br>Spike-23847-R | CTGCCTTTCCAACAATTTGGCA<br>TGTGTACAAAAACTGCCATATTGCA | 60 | 608 |
| 23790-24358 | Spike-23790-F<br>Spike-24358-R | GTGGTGATTCAACTGAATGCAGC<br>GCCAATAGCACTATTAAATTGGTTGG | 60 | 569 |
| 24197-24789 | Spike-24197-F<br>Spike-24789-R | GCGGGTACAATCACTTCTGGT<br>GTGAAGTTCTTTTCTTGTGCAGGG | 60 | 593 |
| 24697-25247 | Spike-24697-F<br>Spike-25247-R | GGGCTATCATCTTATGTCCTTCCCT<br>TTACTATGGCAATCAAGCCAGC | 60 | 551 |
| 25071-25553 | Spike-25071-F<br>Spike-25553-R | CTGGCATTAATGCTTCAGTTGTAAAC<br>GCAAGAAGTGCAACGCCAAC | 60 | 483 |
